# Therapeutic Alternatives in the Management of Late Complications of Surgery for Isolated Coarctation of the Aorta

**DOI:** 10.1055/s-0038-1669435

**Published:** 2018-09-12

**Authors:** Imad Tabry, Michael Rush

**Affiliations:** 1 Section of Cardiac Surgery, Department of Surgery, Holy Cross Hospital, Fort Lauderdale, Florida

**Keywords:** graft interposition for repair of coarctation, recurrence of coarctation, development of anastomotic aneurysms and pseudoaneurysms, endovascular stenting for recurrent coarctation, hybrid approach for repair of late complications, ascending to descending aorta bypass grafting through right thoracotomy off-pump

## Abstract

Despite initial technical success in the treatment of coarctation of aorta, late recurrence and/or development of aneurysms and pseudoaneurysms frequently prompt reintervention. The authors hereby present such a patient whose management required more than a single intervention to treat his complex anatomy, and they discuss the therapeutic alternatives under similar circumstances.

## Introduction

Isolated coarctation of the aorta (CO-A) comprises a significant percentage of congenital heart diseases. 1 It is usually diagnosed and treated when discovered, most often during infancy or childhood, and more rarely in adulthood. Initial surgical repair is then performed through a left thoracotomy and consists of either resection of the stenotic site with end-to-end anastomosis or left subclavian artery (SCA) onlay patch aortoplasty. Less common techniques include Dacron or Goretex (polytetrafluoroethylene) patch angioplasty without resection, or interposition graft when the segment of coarcted aorta is too long to allow resection and end-to-end anastomosis. 2 Since 1982, transcatheter balloon dilatation became an acceptable alternative to surgery, and subsequently balloon expandable endovascular stents were used successfully either primarily or to manage failed percutaneous transluminal angioplasty.

Despite technical success, neither initial surgical intervention nor endovascular stenting (EVS) appears to be curative as late recurrence of coarctation and/or development of aneurysms (ANs) or pseudoaneurysms (PANs) frequently prompt reintervention. 3

We hereby present such a patient whose management required more than a single intervention to treat his complex anatomy, and we discuss the therapeutic alternatives under similar circumstances.

## Case Presentation

**Fig. 1:**
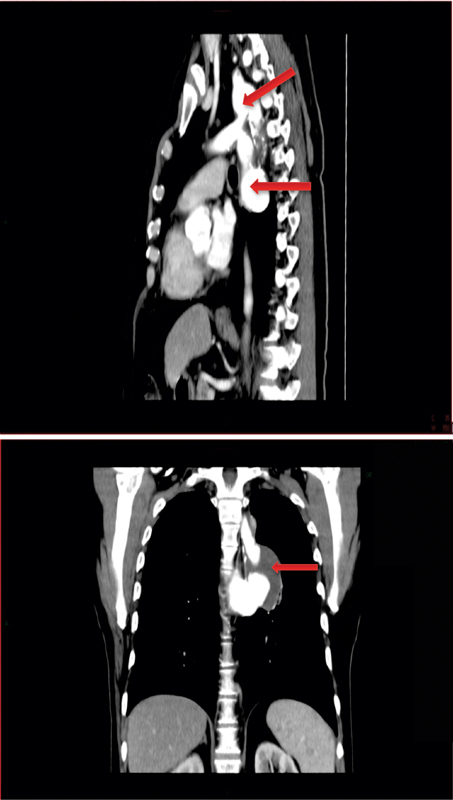
Computed tomographic findings. Upper panel, lateral view: proximal (upper arrow) and distal (lower arrow) large anastomotic pseudoaneurysms of the left subclavian to aorta bypass graft. Lower panel, frontal view: arrow points to large distal graft pseudoaneurysm.

**Fig. 2:**
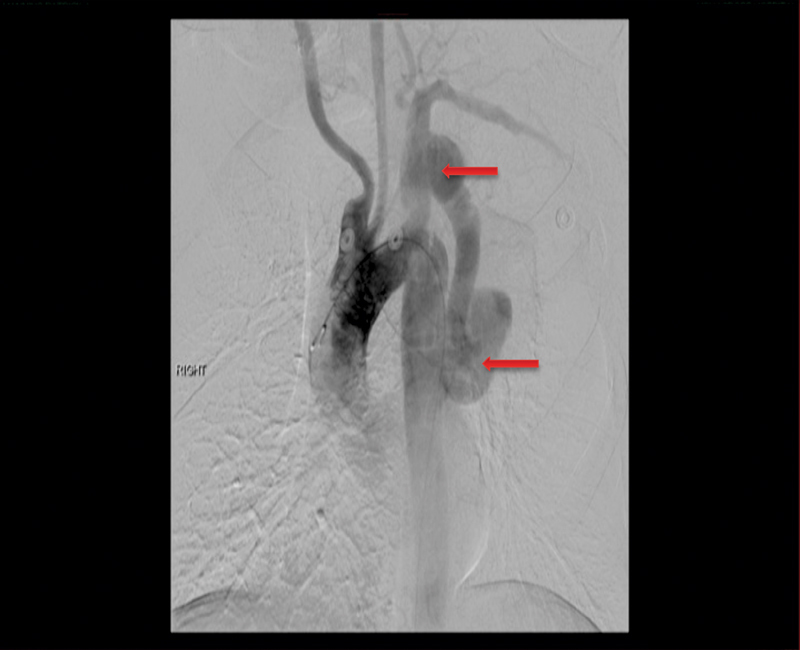
Proximal (upper arrow) and distal (lower arrow) pseudoaneurysms of left subclavian to aorta bypass graft. Occluded right subclavian artery. Bovine origin of carotids. Stenosis of the origin of the left vertebral artery.

A 64-year-old male patient presented to his primary care physician complaining of a dry cough and recurrent bouts of bronchitis as well as frequent orthostatic dizziness. At the age of 20 years, he had undergone repair of CO-A through a left thoracotomy using an interposition Dacron graft between the left SCA and the mid thoracic aorta. His chest radiograph raised suspicion of AN of the thoracic aorta. Computed tomographic angiogram then confirmed the presence of two large anastomotic PANs at both ends of the graft ( Fig. 1 ) as well as an occluded right SCA, stenosis of the left vertebral artery, and bovine origin of the carotid arteries ( Fig. 2 ).

**Fig. 3:**
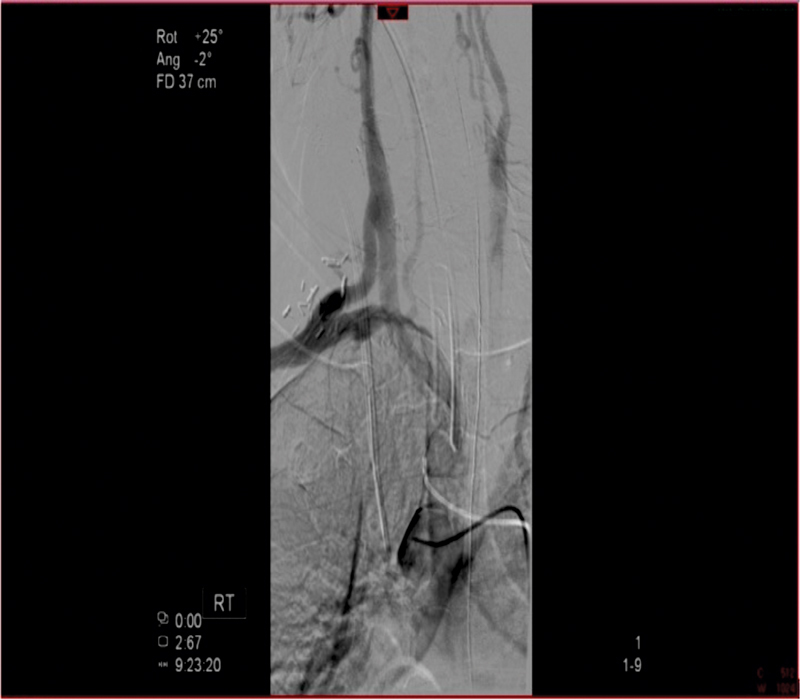
First-stage surgery: right carotid to subclavian artery bypass graft.

**Fig. 4:**
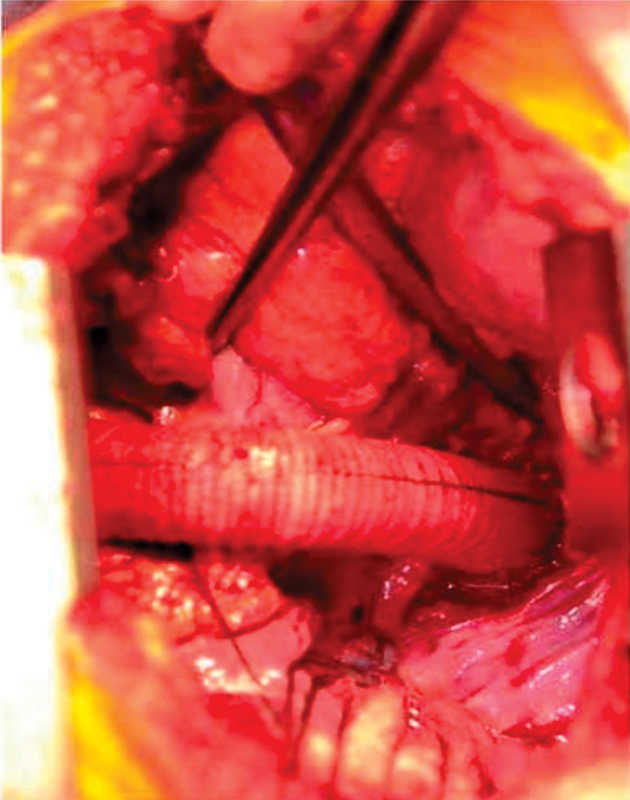
Right posterolateral thoracotomy and off-pump anastomosis of distal graft to supradiaphragmatic aorta.

**Fig. 5:**
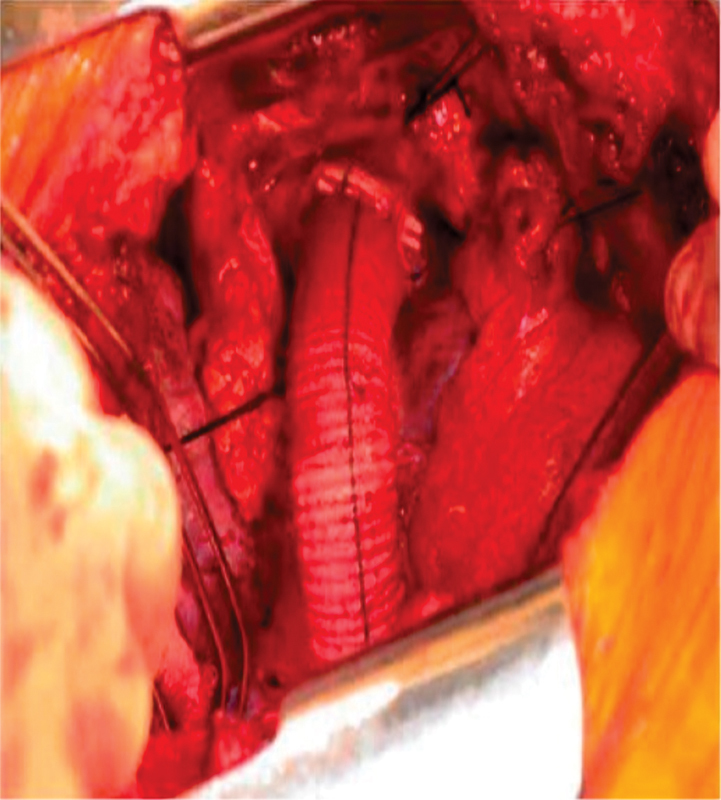
Proximal graft anastomosis to ascending aorta.

**Fig. 6:**
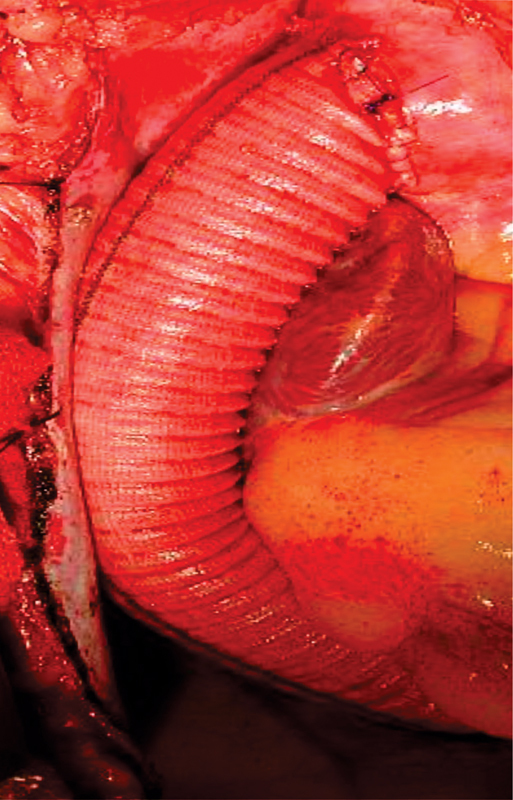
Completed extra-anatomic ascending aorta to descending aorta bypass graft through right thoracotomy and off pump.

In view of this complex anatomy, the decision was made to use a hybrid approach to address these findings. In a first stage, a right carotid artery to right SCA bypass was done using a short segment of Goretex graft ( Fig. 3 ). Next, using single lung ventilation, a right posterolateral thoracotomy was performed and the chest entered through the fifth intercostal space. With the diaphragm retracted inferiorly, the pericardium, posterior to the phrenic nerve, was gently lifted with a long clamp, thus allowing exposure of the distal most portion of the thoracic aorta, medial to the inferior vena cava (IVC). After heparinization, the aorta was partially clamped and a 22 mm Hemashield graft anastomosed in an end-to-side fashion to an appropriate aortotomy using a continuous suture of 5–0 Prolene ( Fig. 4 ). The graft was then brought posteriorly to the IVC and anteriorly to the right hilum, then anastomosed in an end-to-side similar fashion to the partially occluded ascending aorta through a vertical pericardial opening ( Figs. 5 , 6 ).

**Fig. 7:**
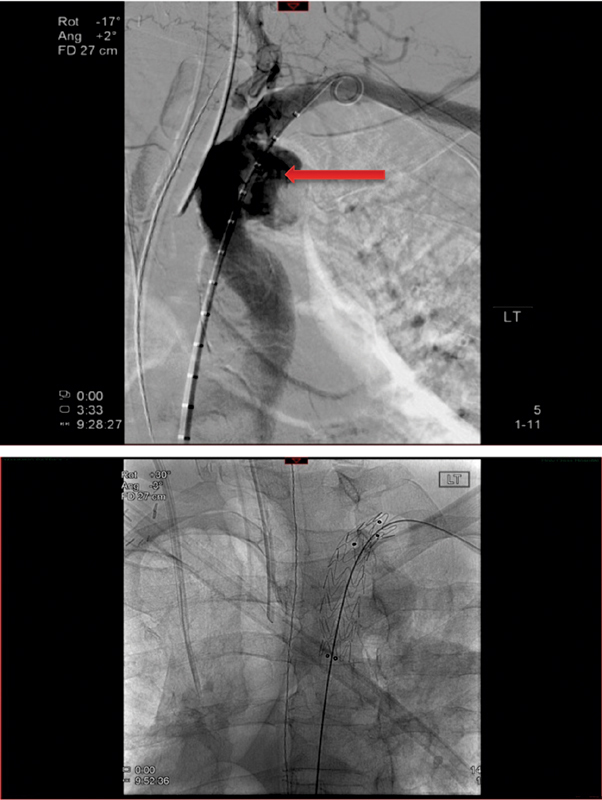
Upper panel: angiographic finding of the proximal anastomotic pseudoaneurysm (arrow). Lower panel: endovascular stenting of the proximal anastomotic pseudoaneurysm with 16 × 80 mm Medtronic covered stent graft deployed across the origin of the left vertebral artery.

**Fig. 8:**
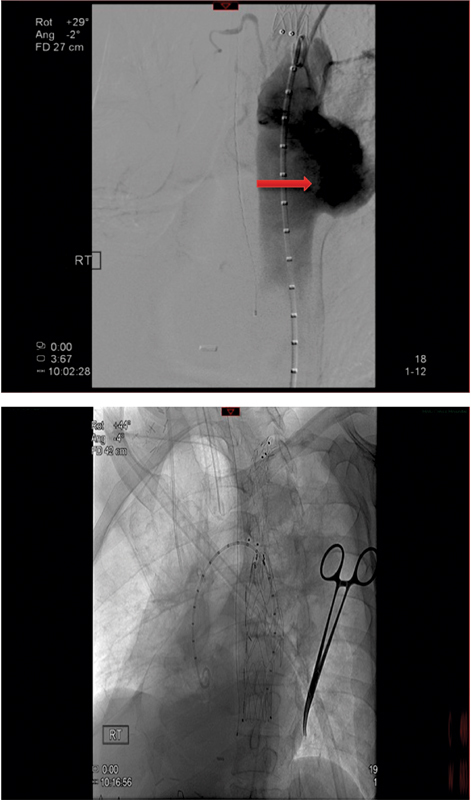
Upper panel: angiographic finding of the distal anastomotic pseudoaneurysm (arrow). Lower panel: endovascular stenting of distal anastomotic pseudoaneurysm with 24 × 100 mm long Medtronic stent graft from level of coarctation to the insertion of the ascending-to-descending thoracic aorta bypass graft.

**Fig. 9:**
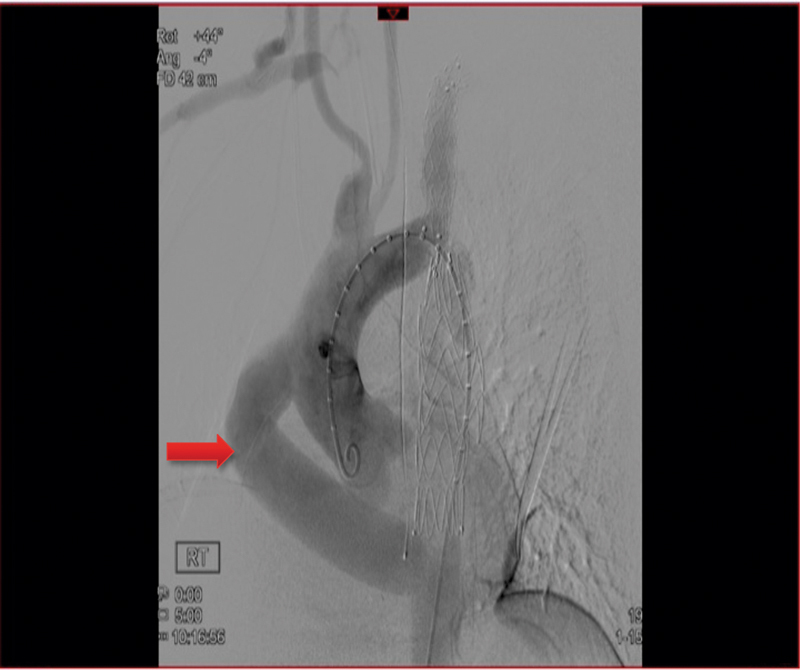
Completion angiogram shows a patent extra-anatomic ascending to descending aorta bypass graft (arrow), occluded left subclavian to thoracic aorta bypass graft, and occluded proximal and distal pseudoaneurysms.

The immediate postoperative course was uneventful, and 5 days later, the patient was brought to the hybrid operating room for completion angiogram. This confirmed patency of the extra-anatomic graft as well as the presence of a 4 cm proximal left SCA anastomotic PAN with close extension to the left vertebral artery origin, and a 4.5 cm distal anastomotic PAN. EVS of the proximal PAN was done using a 16 × 80 mm covered Medtronic stent deployed across the origin of the left vertebral artery ( Fig. 7 ).The distal PAN was excluded using a 26 × 100 mm covered Medtronic stent extending from the level of the coarctation to the insertion of the extra-anatomic bypass graft ( Fig. 8 ). Completion angiogram confirmed patency of the latter and occlusion of the left SCA to thoracic aorta graft as well as of both anastomotic PANs ( Fig. 9 ). The postoperative course was uneventful and the patient remains asymptomatic 2 years later and will be followed up on a yearly basis.

## Discussion

Late complications after surgical repair of CO-A are not uncommon, the most frequent being recurrence of the coarctation and development of anastomotic ANs and PANs. The latter are the result of suture line disruption between a prosthetic patch—used for aortic augmentation—or a tube graft and the aorta, or of the progressive occurrence of fusiform widening of the aorta. There is also an increased risk of such complications after balloon angioplasty, usually occurring early after the intervention, in contrast to their late occurrence after surgical repair of the CO-A.

Left alone, recurrent CO-A ultimately leads to severe hypertension with its associated significant complications. Similarly, conservative management of AN and PAN complicating surgery for CO-A can also be fatal due to a documented high risk of rupture. 4 5 6 Thankfully, several therapeutic options have become available in the prevention and management of such complications.

With increasing patient demand for less invasive options and the development of sophisticated percutaneous technologies, it is now well established that stent implantation carries lower morbidity and mortality compared with surgery for recurrent isolated CO-A. This is particularly so in high-risk patients with coronary artery disease, ventricular dysfunction, or other significant comorbidities. An additional advantage of EVS is the fact that it requires a much shorter hospitalization, causes less postprocedure pain, and allows earlier return to full activities. EVS has also recently emerged as the least invasive approach to treat thoracic aortic ANs and dissections and has thus also grown in popularity in the treatment of anastomotic AN and PAN after CO-A repair, either primarily 7 or in combination with surgical repair, the so-called hybrid solution. 8 9 10 Despite very encouraging early results, there remain, however, several early and late complications of endovascular interventions, 11 namely stent migration, aortic rupture and, at times, difficulty achieving a satisfactory proximal seal without occluding the left SCA. Although often well tolerated, occlusion of this artery may occasionally cause limb ischemia necessitating reimplantation of the left SCA or a carotid to subclavian bypass graft. Interestingly, somatic growth in children with coarctation frequently necessitates further vascular interventions, thus limiting the indication for stenting at a young age.

Surgical reintervention, when indicated, is more challenging. Despite improvements in surgical techniques, it carries a significant morbidity and mortality, particularly when traditional redo left thoracotomy is the chosen approach as this involves the difficult tasks of releasing extensive lung adhesions while avoiding bleeding and major air leaks, mobilizing the thoracic aorta extensively to cross-clamp it above and below the AN, and avoiding phrenic and recurrent laryngeal nerves as well as thoracic duct injuries and preventing the rare occurrence of a devastating spinal cord ischemia. For all those reasons, most surgical reinterventions today avoid left chest re-entry and, instead, aim at creating an extra-anatomic ascending aorta to descending aorta (EA AS-DA) bypass graft. 12 13 This is usually achieved through a median sternotomy and cardiopulmonary bypass, thereby facilitating elevation of the heart and completion of the distal anastomosis to the supradiaphragmatic thoracic aorta through a posterior retrocardiac pericardial rent. This approach has the advantage of also addressing the management of more proximal aortic arch anomalies as well as the repair of coexistent intracardiac anomalies. Less frequently, and using the same sternotomy approach extending to the upper abdomen and without the need for cardiopulmonary bypass, the extra-anatomic graft is extended from the ascending aorta to the supraceliac abdominal aorta. 14 Although achieving excellent results with low morbidity and mortality, the sternotomy approach does not address the issue of AN and PAN complicating coarctation repair. It is indicated mostly in cases of recurrent CO-A with or without concomitant intracardiac pathology as well as in acquired coarctation after repair of traumatic transection of the descending aorta.

A valid alternative approach to tackle recurrent CO-A without concomitant intracardiac pathology is through an off-pump right posterolateral thoracotomy 15 16 17 as described in our case report. Through virgin territory and with the beating heart untouched, an EA AS-DA bypass graft can easily be sutured in place, thus avoiding both sternotomy and cardiopulmonary bypass with their many potential complications.

Unfortunately, both surgical approaches, sternotomy and right thoracotomy alone, fail to address the issue of post coarctation repair of AN and PAN. Because there is no single recipe for the management of these complex cases, the selection of the appropriate technique of repair in each patient's case requires careful evaluation by a multidisciplinary team and a tailored approach. Thus arose the need in our case for a hybrid approach combining both an EA AS-DA bypass around the recoarctation and an EVS of the aneurysmal dilatation or PAN formation as in our case report.

## Discussion

While initial surgical repair of CO-A in infancy remains the gold standard, reintervention at a later age is often necessary due to the frequent recurrence of the coarctation and/or the formation of ANs or PANs secondary to delayed suture line disruption.

Presently, it appears that EVS is ideally suited—and very successful—in most cases of isolated re-CO-A and in some cases of AN or PAN formation. 7 18 In more complex situations, surgical intervention becomes necessary, whether by sternotomy—particularly when there are concomitant arch or intracardiac anomalies that need to be corrected simultaneously—or right thoracotomy, with creation of an EA AS-DA bypass graft. Irrespective of the surgical approach, surgery alone often fails to address the associated AN or PAN. In these cases, a hybrid approach combining both surgery and EVS in a single or staged approach offers a significant advantage in converting an incomplete and risky surgical procedure into a safe and effective one as this case report demonstrates.

There are currently no clear guidelines and no single method to deal with the management of the late complications of surgery for isolated CO-A. Thus, careful evaluation of each case by a multidisciplinary team should allow the selection of the most appropriate therapeutic approach: EVS in most cases of isolated recurrence of the coarctation, surgical approach in more complex cases, or a combination of both, the hybrid approach. Irrespective of the type of repair, and even after successful intervention, all patients undergoing CO-A repair will need lifelong clinical and imaging follow-up to detect and treat potential late complications, not the least being hypertension, congestive heart failure, and coronary artery disease occurrence.
